# Increasing demand for cataract surgery: lessons from a systematic review

**Published:** 2022-12-16

**Authors:** Stevens Bechange, Bhavisha Virendramumar, Elena Schmidt

**Affiliations:** 1 Senior Research Advisor: Sightsavers Uganda Country Office, Kampala, Uganda.; 2 Research Associate Evidence Synthesis: Sightsavers United Kingdom, Haywards Heath, UK.; 3 Director of Evidence, Research and Innovations: Sightsavers United Kingdom, Haywards Heath, UK

**Research provides hints but no solid evidence about how to increase demand.**

**Figure:**
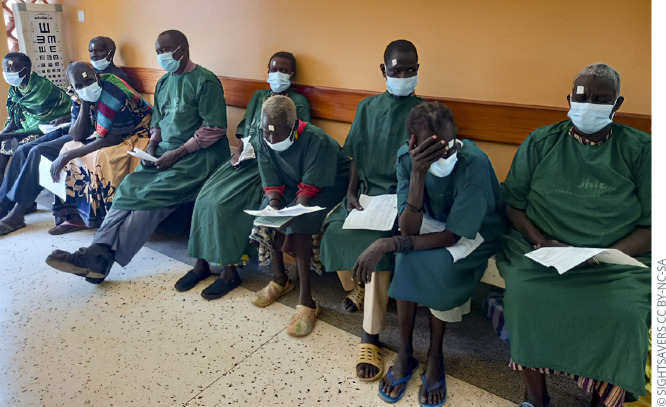
Patients wait to undergo cataract surgery. **UGANDA**

Stimulating demand for surgery is critical for maximising the effectiveness and efficiency of cataract services, as explained elsewhere in this issue. Programmes that do not manage to achieve optimum uptake of cataract surgery may be wasting precious resources on patient mobilisation and screening approaches which do not result in sight-restoring surgery.

In 2020, Sightsavers conducted a systematic review^1^ to identify factors associated with the uptake of cataract surgery and strategies to facilitate demand for surgery in low- and middle-income countries. Out of 18,530 records identified through a search of electronic databases, only seven papers met our inclusion criteria. Given the scale of the problem of cataract blindness, this makes it a neglected area of research. Two studies each were conducted in China and India, one each were from Ghana and Madagascar, and one was a multicountry study conducted in Kenya, Bangladesh, and the Philippines. Two of the studies were randomised controlled trials (RCTs) and the rest were observational studies.

The rates of surgery uptake reported in the studies varied greatly, from 14.4% in north-western China to 91.7% in southern India, but some factors were suggested as increasing demand for surgery.

## At the individual level

Uptake of surgery was lower among women and older patients. There may be other factors that are also important in a specific setting, such as educational level or marital status.

## At the community level

The opinions of ‘significant others,’ particularly family and friends, was a potential factor shaping patient decisions. There is a great need for more studies to better understand the role of community-level factors in increasing demand, so that efforts can be made to change cultural norms where these are a barrier.

## At the health system level

There must be a balance between the need for health facilities to generate enough income to pay personnel and buy supplies, whilst reducing financial barriers experienced by patients. Barriers include direct costs (surgical fees) and indirect costs (such as transport costs or loss of income whilst away from home). Reducing these barriers is generally shown to improve the uptake of surgery, although the influence of financial barriers will vary depending on the local context. Factors such as population density and local transport infrastructure will probably affect the relationship between the cost of surgery and people’s uptake of surgery. Therefore, individual hospitals or local health authorities will need to find out what the main barriers are in their area and how to address them in a sustainable way.

Offering outreach services at regular intervals was reported to improve the uptake of cataract surgery, perhaps due to familiarity and the gradual raising of the local population’s awareness about eye health and the benefits of eye care services. The number of studies exploring these relationships remains limited, so there is a need for more operational research in this area.

## What action should result from this review of the published research?

Our review did not identify interventions that are consistently and strongly associated with improved use of cataract services. The main finding was the glaring lack of evidence on the strategies that work in low- or middle-income countries to improve cataract surgery uptake (only two studies, both from China, addressed this question).

The review points to an urgent need for studies that evaluate different approaches to improve the uptake of surgery and their effectiveness in different settings. It is critical that these studies are of sufficient size to be able to examine statistically significant associations between outcomes and patient characteristics (such as gender or age), as this will help us to understand who benefits from these approaches and who does not.

Eye care programmes and non-governmental organisations can look for opportunities to embed studies in programmes and share their findings widely. Analysis of hospital data could be useful for identifying which groups of people (e.g., women or people with disabilities) come forward, and which do not. This could be supplemented by qualitative studies of patients – e.g., interviewing patients or asking them to complete questionnaires. These could be done in partnership with other organisations with experience in qualitative research, such as local training and research institutions.
